# Supplementary material for: Reorganizing the RNA polymerase II complex for replication of an infectious noncoding RNA in vivo
Source: PLoS Pathog. 2026 Apr 30;22(4):e1014200. doi: 10.1371/journal.ppat.1014200 (PMC13152212; doi:10.1371/journal.ppat.1014200)
Supplement: S1 Fig — PSTVd RNA and 35S:GFP plasmid were co-transfected in protoplasts yielded from wild type or the nrpb2–3 hypermorphic mutant. GFP express from plasmid was repressed in the nrpb2–3 mutant, but PSTVd RNA replication was not affected. Scale bar, 50 μm. (PDF) [file ppat.1014200.s002.pdf]

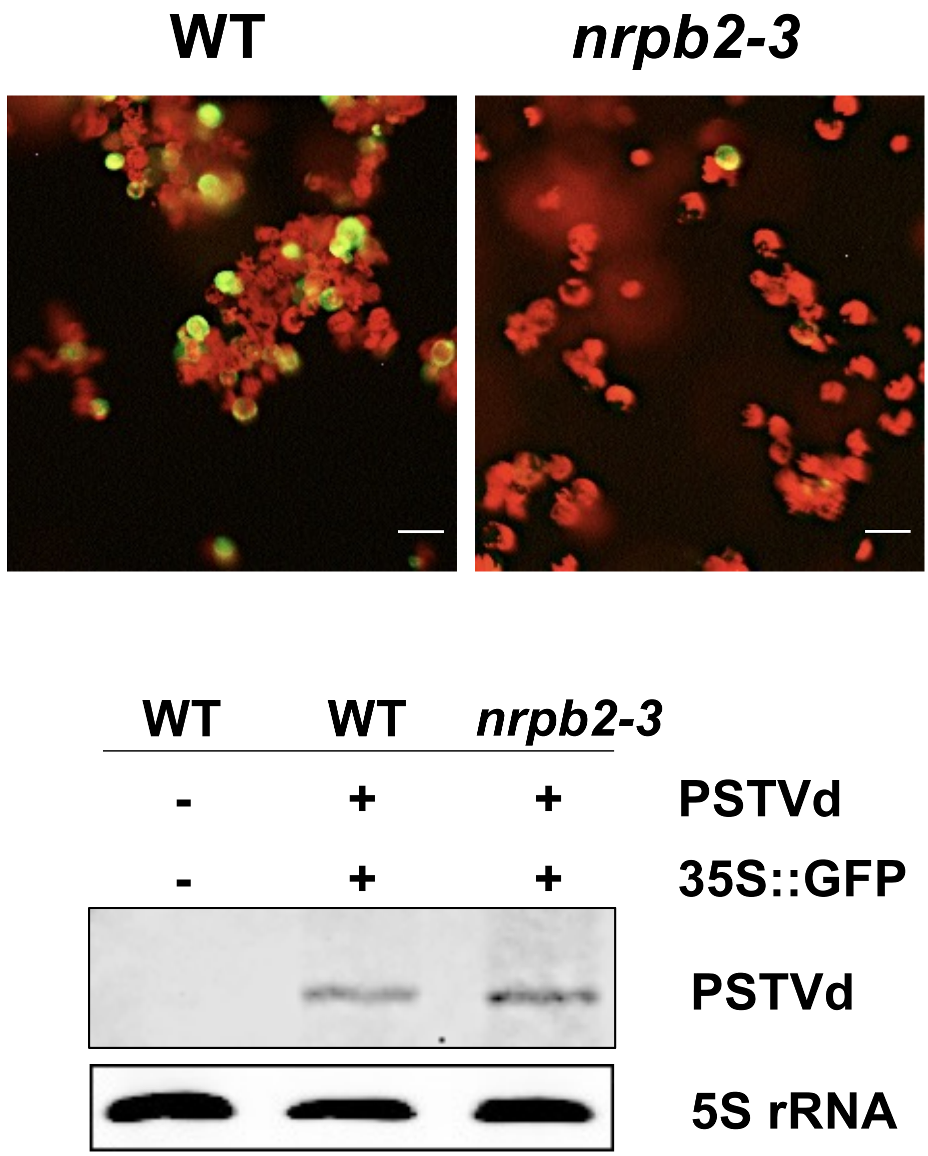

Figure S1. The G435E mutation in Rpb2 affects DNA-dependent transcription but not RNA-templated transcription. PSTVd RNA and 35S:GFP plasmid were co-transfected in protoplasts yielded from wild type or the *nrpb*2-3 hypermorphic mutant. GFP express from plasmid was repressed in the *nrpb*2-3 mutant, but PSTVd RNA replication was not affected. Scale bar, 50 µm.
