# Supplementary material for: Reorganizing the RNA polymerase II complex for replication of an infectious noncoding RNA in vivo
Source: PLoS Pathog. 2026 Apr 30;22(4):e1014200. doi: 10.1371/journal.ppat.1014200 (PMC13152212; doi:10.1371/journal.ppat.1014200)
Supplement: S2 Fig — (PDF) [file ppat.1014200.s003.pdf]

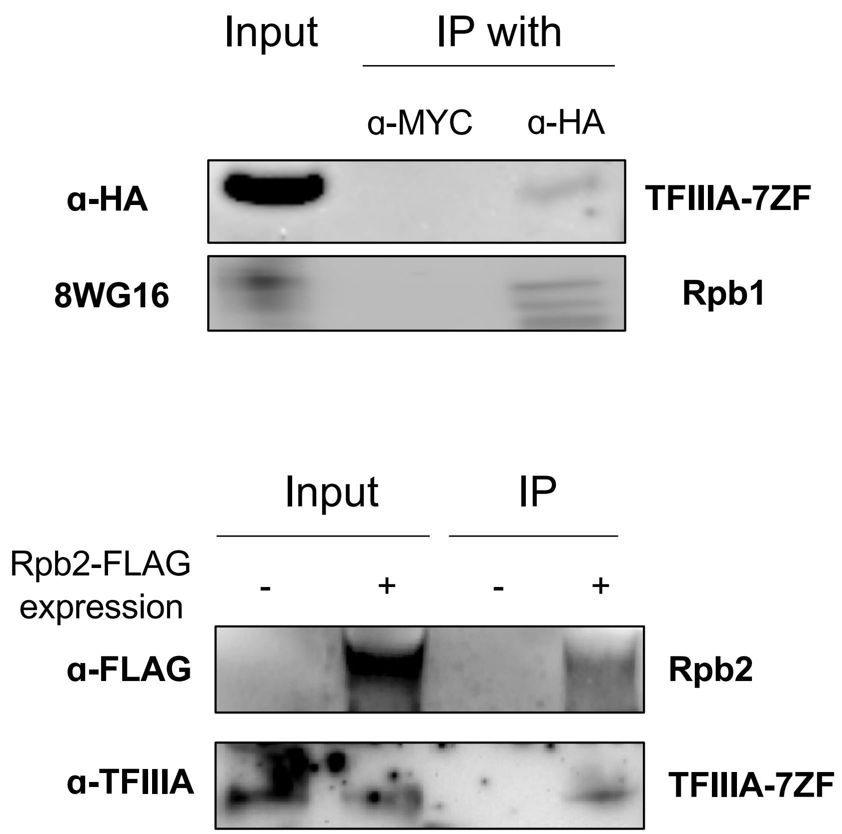

S2 Fig. Co-immunoprecipitation of Rpb1/TFIIIA-7ZF and Rpb2/TFIIIA-7ZF. HA-tagged NbTFIIIA-7ZF was used for immunoprecipitation in the top panel, and Rpb1 was detected using 8WG16. Detailed protocol was described in Ref 13. FLAG-tagged Rpb2 (Ref 7) was used for immunoprecipitation in the bottom panel, and TFIIIA-7ZF was detected using home-made polyclonal antibody (Ref 13).
