# Supplementary material for: Reorganizing the RNA polymerase II complex for replication of an infectious noncoding RNA in vivo
Source: PLoS Pathog. 2026 Apr 30;22(4):e1014200. doi: 10.1371/journal.ppat.1014200 (PMC13152212; doi:10.1371/journal.ppat.1014200)
Supplement: S3 Fig — (PDF) [file ppat.1014200.s004.pdf]

Pol II with DNA template

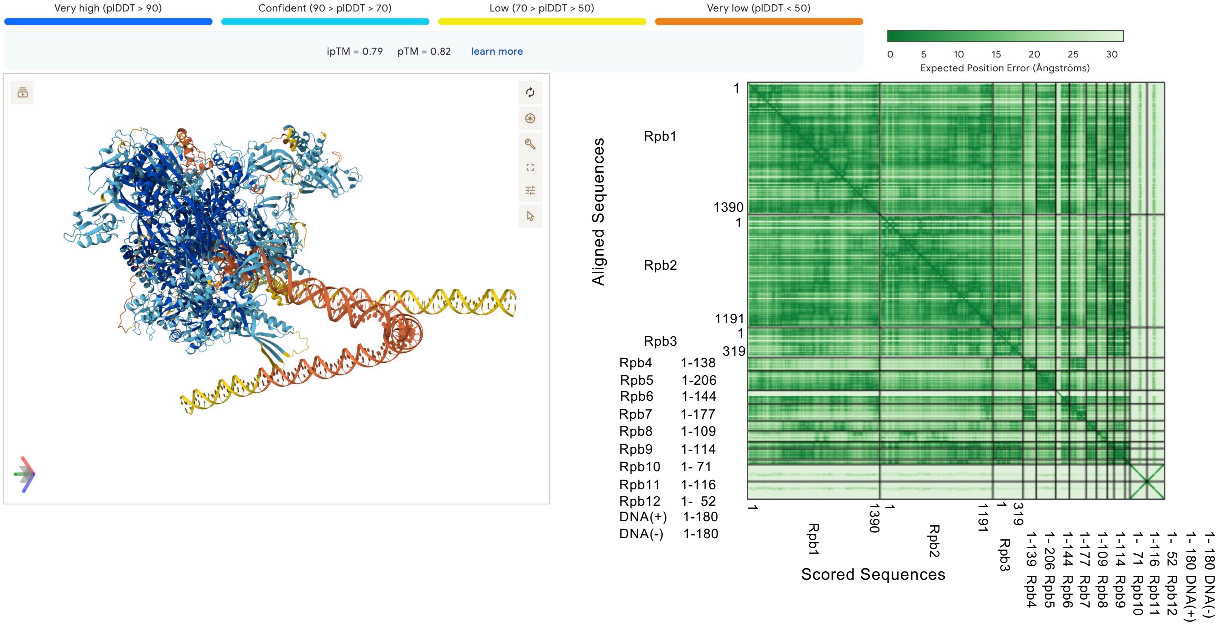

Remodeled Pol II with PSTVd and TFIIIA-7ZF

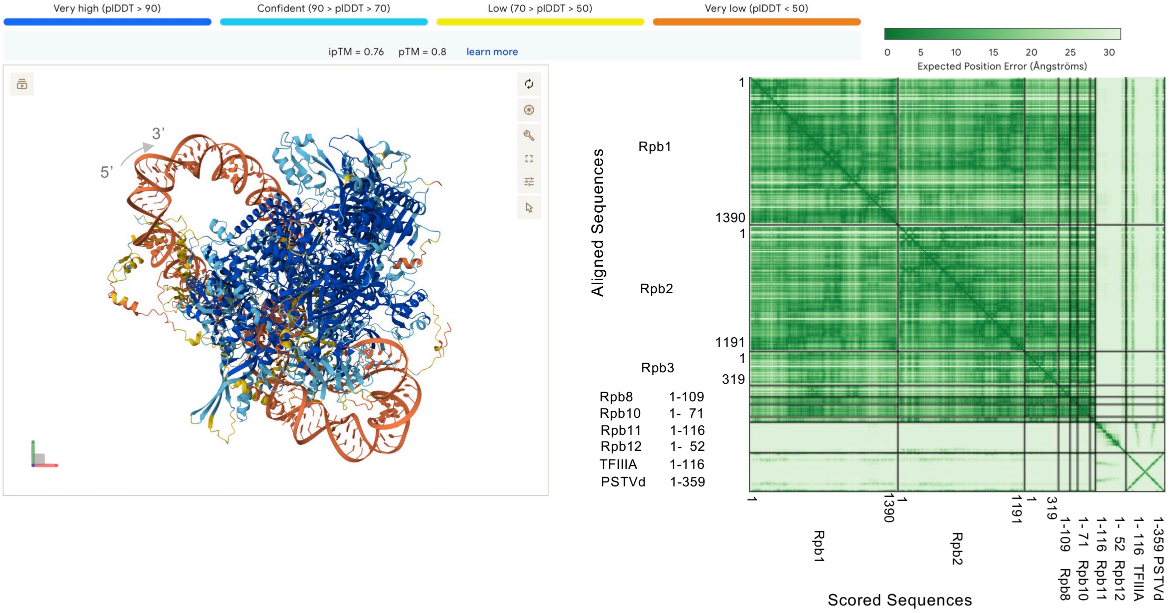

TFIIIA-7ZF and PSTVd

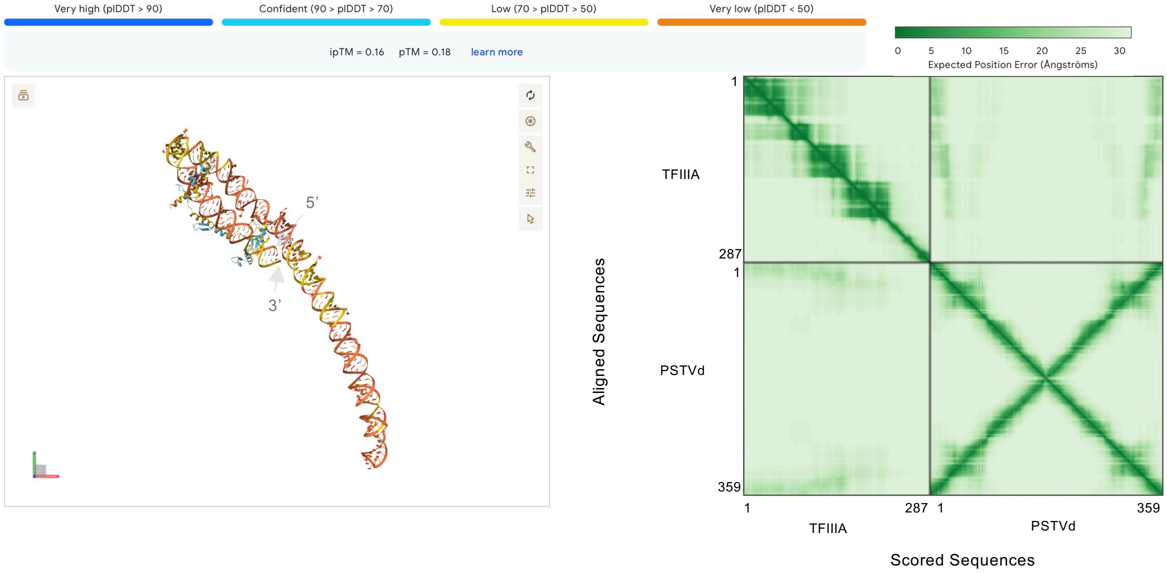

S3 Fig. AlphaFold 3 confidence scores
