# Supplementary material for: Reorganizing the RNA polymerase II complex for replication of an infectious noncoding RNA in vivo
Source: PLoS Pathog. 2026 Apr 30;22(4):e1014200. doi: 10.1371/journal.ppat.1014200 (PMC13152212; doi:10.1371/journal.ppat.1014200)
Supplement: S4 Fig — (PDF) [file ppat.1014200.s005.pdf]

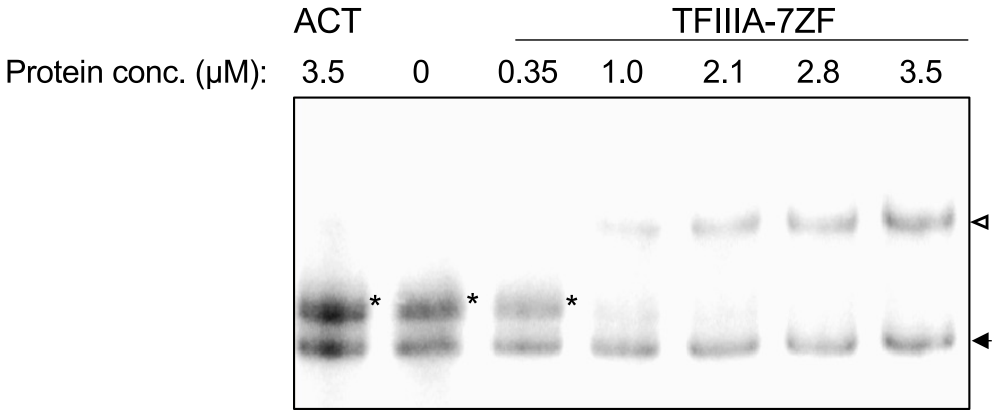

S4 Fig. EMSA with actin protein as a negative control. The solid arrow depicts the position of free RNA substrate. The hollow arrow depicts the position of protein-RNA complexes. * depicts bands formed by alternative RNA conformations (as described in ref 62). About 20 ng unlabeled PSTVd RNA was used as substrate in each lane. The non-radioactive-based EMSA protocol for this data was described in details in ref 62. ACT, actin protein.
